# Irritable Bowel Syndrome/Inflammatory Bowel Disease Overlap: Less Common Than We Think

**DOI:** 10.1016/j.gastha.2024.08.005

**Published:** 2024-08-10

**Authors:** Lauren K. Burdine, Shana Rakowsky, Laurie Grossberg, Loren Rabinowitz, BIDMC IBD Research Center, Kostas Papamichael, Tina Deyhim, Chandler Yap, Adam S. Cheifetz, Sarah Ballou

**Affiliations:** 1 Department of Medicine, Beth Israel Deaconess Medical Center, Boston, Massachusetts; 2 GastroHealth Framingham, Framingham, Massachusetts; 3 Division of Gastroenterology, Department of Medicine, Beth Israel Deaconess Medical Center, Boston, Massachusetts

Inflammatory bowel disease (IBD) is a chronic, immune-mediated condition including ulcerative colitis and Crohn’s disease. Symptoms include abdominal pain and diarrhea as sequelae of underlying mucosal inflammation. Many symptoms of IBD are similar to irritable bowel syndrome (IBS).^1^ Although these conditions are distinct, many patients with IBD report persistent symptoms even after achieving endoscopic remission, highlighting the potential for coexisting IBS in patients with IBD.

Prior literature suggests that 20%–46% of patients with inactive IBD have comorbid IBS,^2^^,^^3^ which is significantly higher than the 4%–11% prevalence of IBS in the general population.^4^ A recent meta-analysis of IBS-type symptoms in IBD reported a pooled prevalence of 32.5%, but 25% when remission was defined by endoscopic or histologic findings.^3^ However, many studies assessing IBS in patients with IBD have been limited by retrospective study design and/or insufficiently stringent methods to confirm IBD remission.^2^^,^^3^^,^^5^^,^^6^

Recent investigations of IBS/IBD overlap have focused on differentiating these entities and ruling out active inflammation in patients with IBD, leading to controversy on whether these conditions mutually coexist or if IBS-type symptoms in patients with IBD are indicative of inflammation.^3^ Determining whether symptoms relate to IBS (in patients with inactive IBD) or inflammation in active IBD is essential to guide treatment. In addition, IBD and IBS have each been associated with impaired quality of life, high rates of psychiatric comorbidities, and sleep disturbance. Patients with IBS/IBD overlap tend to experience a greater burden of these symptoms compared to IBD patients without concomitant IBS.^5^^,^^7^^,^^8^

The aim of this prospective study was to define the prevalence of IBS in IBD patients with quiescent disease and to compare the severity of gastrointestinal (GI) symptoms and psychological distress among those with and without IBS overlap.

Patients at a tertiary care academic medical center were invited to participate at the time of colonoscopy. Eligible patients included individuals 18 years or older with a confirmed diagnosis of IBD. Informed consent was obtained and patients completed the following surveys prior to their colonoscopy: Patient-Reported Outcomes Measurement Information System (PROMIS) questionnaires for constipation, diarrhea, anxiety (Short Form 7a), and depression (Short Form 8a), the Rome IV diagnostic questionnaire, the IBS severity scoring system (IBS-SSS), and the visceral sensitivity index. Inflammatory markers were obtained when available within 90 days of colonoscopy for each patient.

257 patients (51.2% female, 89.5% White, and mean age 49.13 years) participated in the study, of which 125 (49%) were found to be in remission, defined as no visualized endoscopic or histologic inflammation. Eleven patients (7.4% of the patients without active disease) met the full criteria for IBS using the Rome IV criteria. However, this number increased to 19 (15.2%) patients with IBS using validated IBS-SSS cutoff scores representing “moderate-to-severe” symptoms. Patients with inactive IBD who reported moderate to severe IBS symptoms were presumed to have IBS for analytic purposes.

Patients with IBS with inactive IBD (IBS+/IBD) were compared to patients without IBS with inactive IBD (IBS-/IBD) and those with active IBD. Most IBS+/IBD patients (73.1%) were female, and rates of Crohn’s disease, ulcerative colitis, and IBD-unspecified were not significantly different between groups. Rates of biologic use were different between the 3 groups (*P* = .008), with the highest rate of biologic use (78.9%) in IBS+/IBD, compared to IBS-/IBD (42.5%) and active IBD (54.5%).

Patients with IBS+/IBD reported diarrhea severity similar to patients with active IBD and worse than IBS-/IBD (*P* < .001). IBS+/IBD patients also reported more constipation, anxiety, GI-specific anxiety, and depression compared to both active IBD and IBS-/IBD (Table). IBS-SSS scores were significantly higher among IBS+/IBD compared to active IBD (*P* < .001). This comparison was not performed between IBS+/IBD and IBS-/IBD due to having used the IBS-SSS to distinguish between the 2 groups.

**Table:** Clinical and Psychological Characteristics of Patients With Active IBD, IBD in Remission Without IBS Symptoms, and IBD in Remission With IBS Symptoms

Clinical and psychological variables	Active IBD	IBD in remission without IBS (IBS-/IBD)	IBD in remission with IBS (IBS+/IBD)	*P*
	n = 132	n = 106	n = 19	
% female, n (%)	64 (48.50%)	54 (51.40%)	14 (73.70%)	.338
Age (mean [SD])	46.73 (14.26) - a	52.18 (16.17) - b	49.16 (12.79) – ab	**.022**
Diarrhea (PROMIS)	51.77 (9.97) - a	46.87 (8.08) - b	53.89 (11.59) - a	**<.001**
Constipation (PROMIS)	46.15 (8.55) - a	44.49 (6.22) - a	53.72 (7.83) - b	**<.001**
Anxiety (PROMIS)	52.38 (9.25) - a	50.26 (8.90) - a	58.63 (8.13) - b	**<.001**
Depression (PROMIS)	46.65 (7.96) - a	45.79 (7.88) - a	52.44 (9.50) - b	**.005**
Sleep (PROMIS)	52.93 (3.22) -a	53.75 (2.60) -a	52.04 (2.90) -a	**.023**
GI-specific anxiety	28.15 (20.11) - a	18.98 (17.13) - b	48.58 (15.39) - c	**<.001**
IBS-SSS scores	121.07 (98.08) - a	72.84 (49.66) -NA	258.16 (65.36) - b	**<.001**
IBD subtypes				
% with CD, n (%)	67 (50.8%)	40 (37.7%)	9 (47.4%)	.131
Ileal	18 (26.9%)	6 (15.4%)	3 (33.3%)	
Colonic	12 (17.9%)	11 (28.2%)	0 (0.0%)	
Ileocolonic	36 (53.7%)	22 (56.4%)	6 (66.7%)	
Upper	1 (1.5%)	0 (0.0%)	0 (0.0%)	
% with UC, n (%)	56 (42.4%)	59 (55.7%)	8 (42.1%)	.111
Proctitis, n (%)	4 (7.1%)	5 (8.5%)	0 (0%)	
Left sided, n (%)	27 (48.2%)	21 (35.6%)	4 (50%)	
Extensive, n (%)	25 (44.6%)	33 (55.9%)	4 (50%)	
% with IBD-U, n (%)	9 (6.8%)	7 (6.6%)	2 (10.5%)	.827
Medications				
% on biologic	54.5%	42.5%	78.9%	**.008**
% on 5-aminosalicylate	38.6%	45.3%	10.5%	**.016**
% on small molecule^a^	4.5%	1.9%	0.0%	.361
% on immunomodulator^b^	9.1%	13.2%	15.8%	.494
% on steroid	9.1%	0.9%	0.0%	.10
% on no meds	9.2%	9.5%	5.6%	.861
Inflammatory markers				
CRP (n = 84; n = 47; n = 10)	7.60 (16.17)	3.86 (5.47)	3.10 (2.86)	.22
Fecal calprotectin (n = 48; n = 16; n = 5)	782.10 (1209.98)	56.13 (55.76)	37.20 (37.37)	**.029**

PROMIS scores are t-scores (population mean = 50, SD, 10).

IBD in remission with IBS—IBS is defined as scoring at least moderate-to-severe on the IBS severity scoring system.

Bold represents *P* < .05.

Means of continuous variables were compared using analysis of variance and, when significant, post hoc tests for multiple comparisons further explored group differences. Lowercase letters (a,b,c) identify differences between each group. When groups share the same letter, they are not significantly different from each other. When groups have different letters, their means are significantly different from each other.

CD, Crohn’s disease; CRP, C-reactive protein; IBD-U, IBD unspecified; UC, ulcerative colitis.

a Examples of small molecule drugs include: tofacitinib, ozanimod.

b Examples of immunomodulators include: azathioprine, 6-mercaptopurine, methotrexate.

In this well-characterized sample of 125 IBD patients in remission, IBS prevalence was 7% by Rome IV criteria and 15% using a validated IBS severity cutoff—lower than what has been previously reported, yet comparable to documented IBS prevalence in the general population.^3^ Patients with IBS/IBD overlap (IBS+/IBD) reported worse psychological distress and constipation compared to active and inactive IBD and reported similar diarrhea severity to those with active IBD.

This analysis adds to the existing literature that IBS/IBD overlap may be less common than previously reported, particularly when characterizing IBD remission using endoscopic and histologic findings. As there remains heterogeneity in defining endoscopic and histologic remission, future research efforts should be dedicated to developing standardized criteria such that IBD in remission can be differentiated from IBS symptoms as this will aid diagnosis and guide treatment.

These results also indicate that patients with IBD in remission who continue to experience IBS symptoms may present with similar psychosocial profiles (ie, more anxiety, depression, and GI-specific fear and worry) as have been previously reported in the IBS literature. Additionally, biologic use was associated with IBS/IBD dual diagnosis, which may suggest that a history of severe inflammation (such that a biologic would have been required to manage disease) may also be a risk factor for the development of IBS. However, it is unclear if these patients all have a history of objective moderate-to-severe inflammation driving biologic use or if their clinical symptoms prompted their provider to escalate treatment. This issue further highlights the need to understand the differences and potential overlap between IBS and IBD. Regardless, patients with IBS/IBD overlap may benefit from both medical optimization of IBD and IBS and from brain-gut behavioral therapies for IBS such as cognitive behavioral therapy and gut-directed hypnosis.^9^

Our study builds upon the current research of co-occurring IBD and IBS by using objective metrics of disease activity, endoscopic and histologic data, to confirm IBD remission. In doing so, we were better able to distinguish true disease activity. We also more accurately qualified GI and psychological symptoms by utilizing validated patient outcome measures and psychometric screening. Limitations of this study include the modest patient sample size gathered from a single center, as well as underrepresentation of non-White patients. It is also possible that patients with previously established diagnosis of comorbid IBS may be less likely to undergo colonoscopy in the setting of increased GI symptoms and thus may be underrepresented in our sample of patients recruited at the time of colonoscopy. Future research should aim to more fully distinguish between IBD and IBS as clinical entities, as well as work to determine the best interventions for the unique cohort of patients with overlapping IBD and IBS.
